# Tauopathy and Epilepsy Comorbidities and Underlying Mechanisms

**DOI:** 10.3389/fnagi.2022.903973

**Published:** 2022-07-18

**Authors:** Kaylin Hwang, Rahil N. Vaknalli, Kwaku Addo-Osafo, Mariane Vicente, Keith Vossel

**Affiliations:** Mary S. Easton Center for Alzheimer’s Research and Care, Department of Neurology, David Geffen School of Medicine at UCLA, Los Angeles, CA, United States

**Keywords:** tau, epilepsy, mTOR, hyperexcitability, hyperphosphorylation of tau, cognitive decline

## Abstract

Tau is a microtubule-associated protein known to bind and promote assembly of microtubules in neurons under physiological conditions. However, under pathological conditions, aggregation of hyperphosphorylated tau causes neuronal toxicity, neurodegeneration, and resulting tauopathies like Alzheimer’s disease (AD). Clinically, patients with tauopathies present with either dementia, movement disorders, or a combination of both. The deposition of hyperphosphorylated tau in the brain is also associated with epilepsy and network hyperexcitability in a variety of neurological diseases. Furthermore, pharmacological and genetic targeting of tau-based mechanisms can have anti-seizure effects. Suppressing tau phosphorylation decreases seizure activity in acquired epilepsy models while reducing or ablating tau attenuates network hyperexcitability in both Alzheimer’s and epilepsy models. However, it remains unclear whether tauopathy and epilepsy comorbidities are mediated by convergent mechanisms occurring upstream of epileptogenesis and tau aggregation, by feedforward mechanisms between the two, or simply by coincident processes. In this review, we investigate the relationship between tauopathies and seizure disorders, including temporal lobe epilepsy (TLE), post-traumatic epilepsy (PTE), autism spectrum disorder (ASD), Dravet syndrome, Nodding syndrome, Niemann-Pick type C disease (NPC), Lafora disease, focal cortical dysplasia, and tuberous sclerosis complex. We also explore potential mechanisms implicating the role of tau kinases and phosphatases as well as the mammalian target of rapamycin (mTOR) in the promotion of co-pathology. Understanding the role of these co-pathologies could lead to new insights and therapies targeting both epileptogenic mechanisms and cognitive decline.

## Introduction

Tau is a microtubule-associated protein encoded in humans by the microtubule-associated protein tau gene, *MAPT*, on chromosome 17 (Wang and Mandelkow, 2016). In the brain, tau is most abundant in neurons, including neuronal axons, somatodendritic compartments, and nuclei, but it is also present in glia and, to a lesser degree, extracellularly (Morris et al., 2011; Wang and Mandelkow, 2016). The functions of tau in the brain are multifaceted, but its most well-characterized role is in microtubule binding and assembly. Tau is natively unfolded and highly soluble, thus exhibiting little tendency for aggregation. However, under pathological conditions, the hyperphosphorylation of tau reduces its affinity for tubulin and is thought to drive abnormal aggregations of phosphorylated tau (p-tau), such as neuropil threads or neurofibrillary tangles (NFTs), resulting in tauopathies (Wang and Mandelkow, 2016; Kovacs, 2017).

Endogenous tau is also implicated in neuronal activity (Wang and Mandelkow, 2016), though this role of tau is less well understood. Neuronal excitation, in turn, also regulates tau by promoting extracellular release and phosphorylation. Rapid and persisting increases in extracellular tau following *in vivo* (Yamada et al., 2014) or *in vitro* (Pooler et al., 2013) neuronal stimulation suggest that tau amplification is associated with pathological neuronal activation. Given that seizure and chronic epilepsy animal models result in prolonged tau phosphorylation (Liang et al., 2009; Liu et al., 2016; Alves et al., 2019), a growing body of research is examining the role of pathological tau in epilepsy and mechanisms underlying epilepsy and tauopathy comorbidities.

In Alzheimer’s disease (AD), which is the most common tauopathy, an estimated 60% of patients have seizures and subclinical epileptic activity (Vossel et al., 2016, 2017; Lam et al., 2020; Horvath et al., 2021). Seizures are more common in AD and dementia with Lewy bodies than in primary tauopathies, such as frontotemporal dementia and progressive supranuclear palsy (Beagle et al., 2017). However, the possibility of seizures and hyperexcitability in primary tauopathies should not be ruled out, as they occur more frequently in these diseases than in the general population (Beagle et al., 2017; Sanchez et al., 2018). Myoclonus, a sign of network hyperexcitability, is observed in a subset of patients with corticobasal degeneration (Armstrong et al., 2013), and epileptic activity is present in the FTDP-17 animal model of frontotemporal dementia with parkinsonism (Garcia-Cabrero et al., 2013).

Furthermore, tau pathology is repeatedly found in human epilepsy (Sanchez et al., 2018). In a post-mortem series of 138 refractory epilepsy cases of diverse causes, Braak staging of NFTs in the age group 40–65 years revealed increased Braak stages III/IV compared with data from an age-matched series of non-epilepsy cases (Thom et al., 2011). Abnormally high total tau and p-tau levels were also detected in cerebrospinal fluid of status epilepticus patients, with increased total tau correlating with greater risk of developing chronic epilepsy (Monti et al., 2015). Given that neurodegenerative conditions characterized by hyperphosphorylated tau aggregations exhibit increased rates of epilepsy, epilepsies are being re-conceptualized within a tauopathy context (Xi et al., 2011; Sanchez et al., 2018; Ali et al., 2019).

**FIGURE 1:**
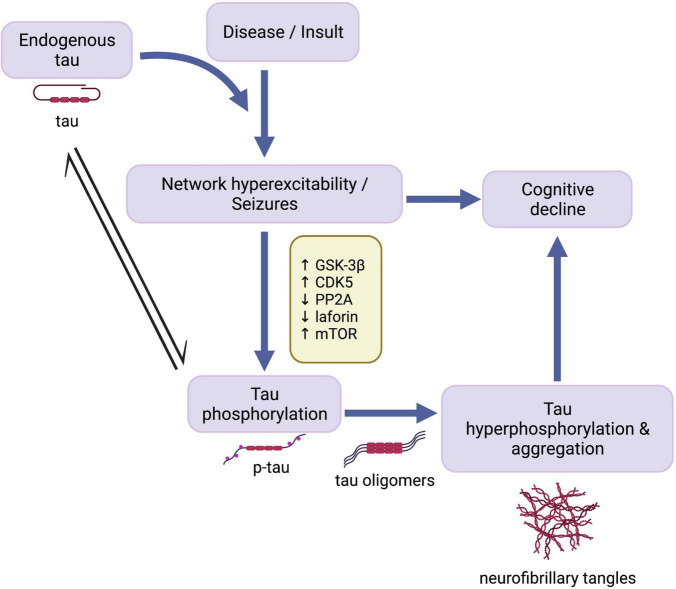
Cascade of events in development of seizures and tau pathology. Endogenous tau has an enabling function in the development of seizure activity following disease onset or traumatic insult. Network hyperexcitability in turn leads to cognitive decline and the activation of mechanisms involving mTOR and tau kinases and phosphatases, resulting in abnormal phosphorylation of tau. Overactivation of these cell signaling pathways increases susceptibility to pathological tau hyperphosphorylation and aggregation, which may also contribute to epilepsy-associated cognitive decline. GSK-3β, glycogen synthase kinase-3β; CDK5, cyclin-dependent kinase 5; PP2A, protein phosphatase 2A; mTOR, mammalian target of rapamycin; p-tau, abnormally phosphorylated tau. Created with BioRender.com.

As such, the present review seeks to explore the following three main questions: (1) Does tau play a role in mediating network hyperexcitability and seizure activity across different epilepsy disorders? (2) Do comorbid tauopathies and epilepsies stem from independent or common mechanisms? (3) How do tauopathy and epilepsy comorbidities contribute to disease-related cognitive impairment? In light of evidence indicating tau-mediated epileptic activity and dysregulation of tau-related cell signaling pathways across seizure disorders, we propose a potential overarching mechanism (Figure 1) whereby endogenous tau helps enable network hyperexcitability, which triggers homeostatic responses aimed, in part, to disable tau activity by phosphorylation. Resulting tau hyperphosphorylation and aggregation may in turn further contribute to cognitive impairments seen in some seizure disorders.

## Role of Tau in Epileptic Activity

Across various animal models that exhibit epilepsy, reducing tau levels reduces network hyperexcitability, seizure severity, and latency to seizure stages (Roberson et al., 2007; Ittner et al., 2010; DeVos et al., 2013; Holth et al., 2013; Wang and Mandelkow, 2016; Ali et al., 2019). Endogenous levels of tau also positively correlate with chemically induced seizure susceptibility in wild-type mice (DeVos et al., 2013), suggesting a role of endogenous tau in mediating epileptic activity.

Though not a primary epilepsy, autism spectrum disorder (ASD) is a disease with links to both seizures and tau. Several studies reveal a strong relationship between epilepsy in individuals with autism and autism in those with epilepsy (Viscidi et al., 2013; Reilly et al., 2015; Sundelin et al., 2016), with the prevalence of epilepsy in ASD doubling in adolescence (26%) compared to childhood (12%) (Hara, 2007; Woolfenden et al., 2012; Sharma et al., 2021). Likely explanations supporting the conjugation of the two conditions include an imbalance in neuronal excitation/inhibition (Bourgeron, 2009; Nelson and Valakh, 2015; Specchio et al., 2022). In the *Cntnap2^–/–^* mouse model of autism with focal epilepsy, global tau knockdown prevents epileptic activity in addition to other autistic-like behaviors (Tai et al., 2020), indicating an epileptogenic role of tau in ASD.

Genetic ablation of tau, even by 50% by inactivation of a single *Mapt* allele, also reduces epileptic activity, high mortality rates, and cognitive deficits in the *Scn1a* mouse model of Dravet syndrome, a severe and intractable childhood epilepsy that is caused by mutations in the *SCN1A* gene and can lead to autism (Catterall et al., 2010; Li et al., 2011; Gheyara et al., 2014; Anwar et al., 2019; Tai et al., 2020). This effect also occurs in the *Scn1a* model following postnatal injection of tau-targeting antisense oligonucleotides (Shao et al., 2022), suggesting that antisense oligonucleotides may be a promising treatment avenue for children with Dravet syndrome. Shao et al. (2022) further found that selective genetic ablation of tau in hippocampal excitatory neurons but not in inhibitory neurons mediates the neuroprotective effects of tau reduction in the *Scn1a* model. The authors propose that the suppression of epileptic activity by tau reduction may therefore result from a lower hypersynchrony of excitatory neuronal activity rather than greater inhibitory regulation.

In some models, pathological tau, rather than endogenous tau, can contribute to epileptic activity. Temporal lobe epilepsy (TLE) is one of the most prevalent forms of focal epilepsy (Tellez-Zenteno and Hernandez-Ronquillo, 2012; Asadi-Pooya et al., 2017), and in the electrical amygdala kindling rodent model of TLE, tau-knockout mice do not differ from wild-type mice in seizure outcome following repeated kindling (Liu S. et al., 2017). However, kindling produces longer epileptic discharge durations and accelerated seizure progression in rTg4510 transgenic mice, which overexpress P301L tau in forebrain and develop increased p-tau and NFTs (Liu S. et al., 2017). These findings suggest that an increase in p-tau and tau aggregation promotes kindling-induced epileptogenesis.

Taken together, findings across different seizure disease models reveal a significant function of endogenous tau, as well as pathological tau, in the mediation of epileptic activity. Given tau’s role in modulating neuronal activity under normal physiological conditions (DeVos et al., 2013; Chang et al., 2021), endogenous tau likely contributes to network hyperexcitability across primary and secondary epilepsies. In humans, tau mRNA expression and protein levels in the brain can vary greatly (Trabzuni et al., 2012). And though exact reasons for individual differences in endogenous tau levels remain unknown, high levels may consequently predict a person’s susceptibility to epileptic activity. Elevated tau measurements in cerebrospinal fluid have in fact been shown to correlate with seizure type and duration in patients with epilepsy (Tumani et al., 2015). Higher levels of endogenous tau alone may not cause seizures, but it is possible that this may predispose an individual to seizure development upon pathogenesis.

## Presence of P-Tau Pathology in Seizure Disorders and Links to Epileptic Activity

While levels of endogenous or total tau differ across examinations of patients with epilepsy, increased levels of p-tau in the brain are found across many seizure disorders (Thom et al., 2011). These include patients with TLE, Dravet syndrome, Nodding syndrome (Thom et al., 2011; Pollanen et al., 2018; Hotterbeekx et al., 2019), Niemann-Pick type C disease (NPC) (Auer et al., 1995; Love et al., 1995; Suzuki et al., 1995; Malnar et al., 2014), focal cortical dysplasia IIB (FCDIIb) (Sen et al., 2007; Iyer et al., 2014), and tuberous sclerosis complex (TSC) (Sarnat and Flores-Sarnat, 2015; Liu et al., 2020), as well as animal models of post-traumatic epilepsy (PTE) (Cho et al., 2020; Alyenbaawi et al., 2021), Lafora disease (*Epm2a^–/–^*) (Ganesh et al., 2002; Puri et al., 2009; Machado-Salas et al., 2012), and ASD (Gassowska-Dobrowolska et al., 2021). It should be noted that tau aggregation in the brain is associated with older age and is generally uncommon in healthy young adults (Braak et al., 2011; Crary et al., 2014). However, the presence of tau pathology in childhood or adolescent-onset epilepsies, such as Dravet syndrome, Nodding syndrome, Lafora disease, NPC, TSC, and ASD, and in relatively younger TLE patient cohorts (Puvenna et al., 2016; Smith et al., 2019; Gourmaud et al., 2020) suggest a causal link between seizure activity and p-tau accumulation.

In animal models of TLE, tau hyperphosphorylation is observed in relevant brain regions, including the amygdala, hippocampus, and cortex, following chemical and electrical amygdala kindling (Jones et al., 2012; Liu et al., 2016; Alves et al., 2019). And in humans with chronic epilepsy, elevated p-tau is present in post-mortem (Thom et al., 2011) as well as surgically resected tissue (Puvenna et al., 2016; Tai et al., 2016; Liu C. et al., 2017; Prada Jardim et al., 2018; Smith et al., 2019; Gourmaud et al., 2020). For instance, analysis of resected temporal lobe tissue by Tai et al. (2016) found pathological tau phosphorylation in the form of neuropil threads, NFTs, and pre-tangles in 31 of 33 TLE patients between 50 and 65 years of age. Interestingly, observations of subpial bands formed by cortical p-tau depositions have been made across separate studies (Puvenna et al., 2016; Tai et al., 2016; Smith et al., 2019), providing evidence for a novel pattern of tau pathology in TLE that may result from seizure-induced reorganization of temporal lobe networks (Tai et al., 2018).

Tau hyperphosphorylation is also consistently present in the initial and long-term secondary mechanisms initiated by traumatic brain injury (TBI) (Petraglia et al., 2014; Zheng et al., 2014; Rubenstein et al., 2015; Ali et al., 2019; Tan et al., 2020), a leading cause of morbidity and mortality worldwide (Hay et al., 2016). For instance, sustaining even a single TBI can result in progressive NFT formation that is more extensive and severe than what is expected with normal aging (Johnson et al., 2012; Zanier et al., 2018). Furthermore, it is estimated that over 50% of severe TBI cases will result in seizures or PTE (Kovacs et al., 2014), and animal models reveal increased p-tau levels in the brain associated with TBI-induced epileptic activity (Cho et al., 2020; Alyenbaawi et al., 2021). In a recent study, Alyenbaawi et al. (2021) presented a novel model of transgenic zebrafish expressing a fluorescent tau biosensor where TBIs by blast-like pressure waves induced progressive tauopathies. Tau aggregation positively correlated with TBI severity and the presence of seizure-like clonic shaking. Furthermore, tau aggregation following TBI administration was prevented by the anti-convulsant ezogabine and exacerbated by kainate treatment, demonstrating a role of seizure activity in mediating tauopathy development (Alyenbaawi et al., 2021).

A mechanism by which epileptogenesis gives rise to tau hyperphosphorylation may underlie the high incidence of tauopathy and epilepsy co-pathology that is found in diseases such as AD and dementia with Lewy bodies (Vossel et al., 2016, 2017; Beagle et al., 2017). Given that endogenous tau plays a role in regulating neuronal activity, disruption in the homeostatic balance of tau modifications may mediate seizure comorbidities observed in these diseases, and epileptic activity may in turn help drive tau hyperphosophorylation. In classical tauopathies where overt epilepsy infrequently occurs, tau hyperphosphorylation can arise from a variety of different causes (Kovacs, 2017). However, it is also possible that epileptic activity in these tauopathies is clinically underrecognized due to being non-motor or subclinical in nature, as suggested by the detection of subclinical epileptic activity in over 40% of AD patients during overnight electroencephalography and 1-h magnetoencephalogram recordings (Vossel et al., 2016). More studies involving extended periods of neurophysiological monitoring are therefore required to investigate the presence of epileptic activity and its potential contribution to tau hyperphosphorylation in primary tauopathies.

It should also be noted that tau pathology is not universally found in connection with epileptic activity. For instance, 31% of the post-mortem refractory epilepsy cases studied by Thom et al. (2011) were classified as Braak Stage 0, and analysis of surgically resected tissue from 56 TLE patients by Silva et al. (2021) found p-tau-positive neurons in only two samples. The absence of pathological tau deposition in these cases indicates that epileptogenesis does not always lead to tauopathies. However, the factors that determine the subsequent development of tau pathology in some cases of aberrant network excitability but not others remain unclear. It is possible that the formation of pathological tau deposits is linked to specific seizure disorders or that mechanisms mediating tau hyperphosphorylation are overactivated in cases of more severe epilepsy.

## Dysregulation of Cell Signaling Activity Upstream of Tau Phosphorylation

### Kinases

Given that the balance of tau phosphorylation states is regulated by enzymatic activity, investigations into the impairment of tau kinases and phosphatases in seizure disorders reveal links between epileptic activity and tau hyperphosphorylation. Investigations into novel pharmacological interventions targeting tau hyperphosphorylation in epilepsy have therefore concentrated on inhibiting and enhancing related phosphorylation and dephosphorylation mechanisms, respectively (Zheng et al., 2014; Ali et al., 2019).

One relevant kinase responsible for tau phosphorylation is glycogen synthase kinase-3β (GSK-3β) (Toral-Rios et al., 2020). Upregulation of GSK-3β is found in surgically resected tissue samples from patients with intractable epilepsy (Xi et al., 2009; Liu X. et al., 2017), and GSK-3β overactivation co-occurs with increased p-tau levels in mesial TLE patients (Liu C. et al., 2017). Inhibition of GSK-3β may have dual benefits given that GSK-3β inhibition reduces tau hyperphosphorylation and NFT formation in tau-overexpressing transgenic mice (Noble et al., 2005; Engel et al., 2006; Leroy et al., 2010) and produces anticonvulsant effects against pentylenetetrazol-induced seizures in zebrafish larvae (Aourz et al., 2019). However, the observation of sustained increases in p-tau levels following kainic acid administration being accompanied by only transient increases in GSK-3β activity (Liang et al., 2009) and the lack of effect on hippocampal p-tau by GSK-3β inhibitor pretreatment in the intra-amygdala kainic acid-induced status epilepticus mouse model (Alves et al., 2019) indicate that GSK-3β is not the only kinase responsible for tau phosphorylation following epileptic activity.

Another protein kinase highly implicated in tau phosphorylation is cyclin-dependent kinase 5 (CDK5). Dysregulation of CDK5 signaling can contribute to neurodegeneration, excitotoxicity, and tau hyperphosphorylation (Cruz et al., 2003). As is seen with GSK-3β, CDK5 overactivation is present in resected tissue from refractory epilepsy patients (Xi et al., 2009; Liu X. et al., 2017), and dysplastic cortical neurons in FCD patients express CDK5 aggregations (Sisodiya et al., 2002). Furthermore, progressive activation of CDK5 co-occurs with increasing tau phosphorylation in rodent seizure models (Chen et al., 2000; Liang et al., 2009), indicating significant mediation of seizure-associated tau hyperphosphorylation by CDK5. For example, in the genetic mouse model of NPC, increased activation of CDK5 and its activator, p25, coincides spatially and temporally with tau pathology, and CDK5 inhibition by roscovitine and olomoucine prevents cytoskeletal protein phosphorylation (Bu et al., 2002; Zhang et al., 2004, 2008).

Both GSK-3β and CDK5 play a role in neuronal excitability through involvement in GABAergic and glutamatergic neurotransmission, and inhibiting their activity can affect network activity through various mechanisms (Sen et al., 2008; Jaworski, 2020; Toral-Rios et al., 2020; Banerjee et al., 2021). Therefore, inhibiting these kinases should be approached with caution. For instance, genetic ablation of the CDK5 activator, p35, increases susceptibility to spontaneous seizures in mice (Chae et al., 1997). Tau levels were not measured in this study, but it is possible that the absence of activated CDK5 in this genetic model results in higher levels of dephosphorylated tau that contribute to neuronal hyperexcitability. These considerations highlight the complexity of kinase regulation in the setting of normal activity and hyperexcitable states. Targeting tau rather than upstream kinases may therefore be a more viable intervention option for seizure disorders (Figure 1).

### Phosphatases

In addition to kinase activity, tau hyperphosphorylation associated with seizure activity may also be due to a lack of tau dephosphorylation by tau phosphatases. Following kainic acid administration in mice, biphasic changes in p-tau levels occur, where decreased phosphorylation is first observed within the first 6-h period followed by a gradual 3–5-fold increase until a 48-h endpoint. This progression is accompanied by a corresponding increase and then decrease in the activation of protein phosphatase 2A (PP2A) (Liang et al., 2009), which is estimated to account for 70% of human brain tau dephosphorylation (Wang and Mandelkow, 2016). It is possible that phosphatase activity is initially triggered to offset elevated tau phosphorylation caused by upregulated kinase activity following an epileptic event. For instance, GSK-3β upregulation causes PP2A activation (Wang et al., 2015). However, long-lasting phosphatase downregulation ultimately occurs, as evidenced by decreased PP2A activity paired with increased p-tau levels observed in epileptogenic brain regions following post-kainic acid status epilepticus, amygdala kindling, and fluid percussion injury in rats (Liu et al., 2016).

Similarly, abnormal p-tau in the form of NFTs are also observed in the *Epm2a^–/–^* mouse model (Puri et al., 2009), which replicates many of the features of Lafora disease caused by *EPM2A* mutations, including laforin deficiency, neuronal degeneration, spontaneous epileptic activity, and the development of Lafora bodies (Ganesh et al., 2002). Laforin is another tau phosphatase (Puri et al., 2009), though further research is required to investigate connections between hyperexcitability states and laforin downregulation in other seizure disorders. At least in the *Epm2a^–/–^* model, pathological tau levels are also associated with increased GSK-3β activation (Puri et al., 2009), suggesting that tau hyperphosphorylation is not mediated by the absence of laforin alone in Lafora disease.

Interestingly, the lack of phosphatase activity may also contribute to epileptic activity. Sodium selenate is a specific agonist for PP2A expressing the regulatory B subunit, an essential subunit for tau dephosphorylation by PP2A (Jones et al., 2012; Liu et al., 2016), and shows promise as a potential antiepileptic treatment option. Sodium selenate treatment attenuates seizure activity and tau hyperphosphorylation and accumulation following administration of pentylenetetrazol or kainic acid as well as in the TLE model of amygdala kindling and the fluid percussion injury model of PTE (Jones et al., 2012; Liu et al., 2016). The antiepileptic effects of sodium selenate persist following drug washout in animal TBI models (Liu et al., 2016), highlighting a potential disease-modifying effect of PP2A upregulation by sodium selenate during epileptogenesis when applied in early PTE disease stage.

The mechanisms through which tau dephosphorylation by phosphatase function alleviates epileptic activity remain unclear. Dephosphorylated tau at sufficient levels may be favorable in chronic epileptic states, or phosphatases may participate in independent signaling pathways that abate neuronal hyperexcitability. Regardless, as was proposed with tau kinases, long-term phosphatase inactivation may serve as a homeostatic response aimed at maintaining higher levels of phosphorylated tau and preventing endogenous tau from enabling network hyperexcitability (Figure 1). Taken together, the discussed findings indicate that seizures give rise to disruptions in the intricate balance of tau kinase and phosphatase activity and that the combined effects of kinase upregulation and phosphatase downregulation contribute to progressive tau hyperphosphorylation and accumulation in seizure disorders.

### Mammalian Target of Rapamycin Pathways

**FIGURE 2:**
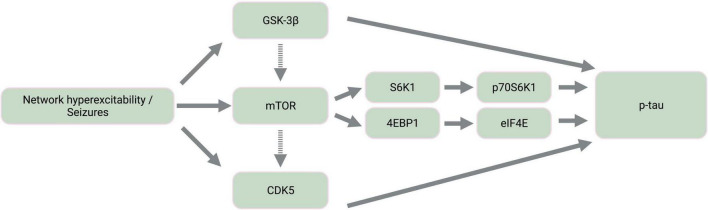
Simplified diagram of activated kinase signaling cascades in epilepsy. Epileptic activity leads to the activation of tau kinases, GSK-3β and CDK5, as well as mTOR. Dashed-line arrows indicate indirect activation of mTOR by GSK-3β through the mTOR complex 1 and of CDK5 by mTOR through amyloid-β aggregation and calpain activation. The downstream targets of mTOR activation involve the activation of additional tau kinases, p70S6K1 and eIF4E. GSK-3β, glycogen synthase kinase-3β; mTOR, mammalian target of rapamycin; CDK5, cyclin-dependent kinase 5; S6K1, ribosomal protein S6 kinase 1; 4EBP1, 4E binding protein 1; p70S6K1, phosphorylated S6K1; eIF4E, eukaryotic translation initiation factor 4E; p-tau, abnormally phosphorylated tau. Created with BioRender.com.

The mammalian target of rapamycin (mTOR) is a highly conserved protein kinase that is implicated in a wide array of cellular and metabolic functions, including cell survival, growth, proliferation, migration, and differentiation (Wong, 2010; Mueed et al., 2018). Activation of mTOR is also a proposed driver of tau pathology given the involvement of tau-related kinases both upstream and downstream of mTOR signaling (Figure 2) and the contribution of mTOR-mediated autophagy dysfunction to tau hyperphosphorylation (Tramutola et al., 2017; Mueed et al., 2018). For instance, the downstream targets of mTOR activation include signaling cascades involving 4EBP1, S6K1, and CDK5, all of which result in tau phosphorylation (Mueed et al., 2018).

Furthermore, mTOR hyperactivation accompanies epileptic activity across different seizure models including animal models of TLE, PTE, FCDII, Dravet syndrome, ASD, and TSC (Meikle et al., 2007; Zeng et al., 2008; Sha et al., 2012; Guo et al., 2013; Gheyara et al., 2014; Butler et al., 2015; Marsan and Baulac, 2018; Tai et al., 2020; Shao et al., 2022). mTOR therefore likely contributes to tau and seizure co-pathology, warranting further pharmaceutical consideration of mTOR inhibition by rapamycin. Rapamycin treatment inhibits both tau hyperphosphorylation (Liu et al., 2013; Ozcelik et al., 2013; Tramutola et al., 2017) and the development of status epilepticus and chronic epilepsy in models of pharmacological seizure induction (Zeng et al., 2008; Huang et al., 2010), TLE (Drion et al., 2016), and PTE (Guo et al., 2013; Butler et al., 2015). Given that active GSK-3β also activates mTOR (Mueed et al., 2018), tau hyperphosphorylation resulting from seizure-associated GSK-3β upregulation may be further exacerbated by GSK-3β-mediated mTOR activation (Caccamo et al., 2013; Figure 2).

**FIGURE 3:**
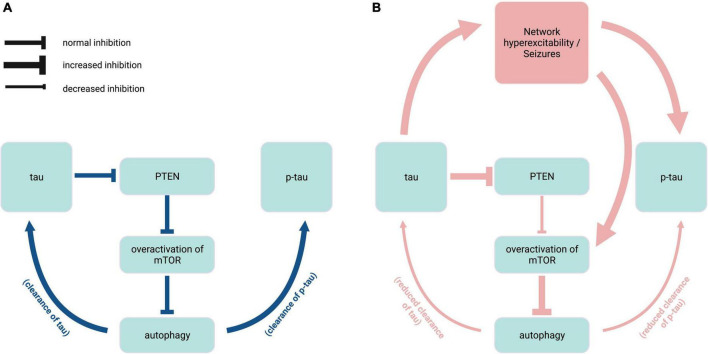
Interplay of endogenous tau, mTOR, and autophagy in epilepsy. **(A)** Under normal physiological conditions (blue), endogenous tau positively regulates mTOR activity via PTEN inhibition, and mTOR in turn negatively regulates autophagy mechanisms that contribute to the clearance of tau and p-tau. **(B)** In hyperexcitability states found in pathophysiological conditions such as tuberous sclerosis complex and Niemann-Pick type C disease (red), overactivation of mTOR due to increased PTEN inhibition causes excess inhibition of autophagy, resulting in reduced clearance of tau species. Elevated levels of normal tau in turn exacerbate epileptic activity and mTOR disinhibition. PTEN, phosphatase and tensin homolog deleted chromosome 10; mTOR, mammalian target of rapamycin; p-tau, phosphorylated tau. Created with BioRender.com.

While the dysregulation of mTOR signaling pathways may manifest differentially across seizure disorders, the many connections between tau and mTOR highlight the significance of maintaining an optimal ratio of dephosphorylated and phosphorylated tau through balanced kinase/phosphatase regulation. Endogenous tau enables mTOR activation through a disinhibition mechanism whereby tau inhibits phosphatase and tensin homolog deleted chromosome 10 (PTEN), which normally inhibits mTOR (Tai et al., 2020; Figure 3). In genetic mouse models of ASD and Dravet syndrome, tau ablation prevents epilepsy and normalizes mTOR overactivation (Gheyara et al., 2014; Tai et al., 2020; Shao et al., 2022), suggesting that reducing tau may be beneficial in these diseases via PTEN disinhibition. mTOR also functions as a negative regulator of autophagy (Tramutola et al., 2017). Therefore, mTOR hyperactivity could prevent clearance of both normal and pathological tau (Chesser et al., 2013). Furthermore, reduced autophagy resulting from mTOR overactivation is implicated in elevated endogenous tau levels in the *TSC2* mouse model of TSC (Caccamo et al., 2013). In seizure disorders such as TSC and NPC that are characterized by autophagy dysregulation (Pacheco et al., 2009; Caccamo et al., 2013), increase in normal tau levels may in turn contribute to both seizure activity and PTEN inhibition, creating a feedback loop of mTOR overactivation that results in further hyperphosphorylation of tau (Figure 3).

## Tau-Associated Cognitive Decline in Epilepsy Disorders

Cognitive impairment is a common comorbidity of seizure disorders. Though cognitive deficits can independently occur in seizure disorders as a direct result of disease etiology, such as trauma, epileptic activity contributes to, and exacerbates cognitive decline (Holmes, 2015). However, there are also investigations into a potential role of tau pathology in epilepsy-associated cognitive decline. While individuals with dementia have higher rates of epilepsy, seizures are experienced more frequently in tauopathy-associated dementias like AD than in other dementias (Sanchez et al., 2018). Cognitive decline is also accelerated in patients who have both AD and seizures compared to those with only AD (Vossel et al., 2017), suggesting that pathological tau and seizures can synergistically worsen cognitive outcomes.

Correlations between tau pathology and cognition are in fact observed in epilepsy. Post-mortem analysis of 138 refractory epilepsy cases revealed that 77% of patients with Braak staging III or higher exhibited progressive cognitive decline (Thom et al., 2011), and increased total and p-tau levels measured in surgically resected tissue from TLE patients are inversely correlated with cognitive scores (Kandratavicius et al., 2013; Tai et al., 2016; Gourmaud et al., 2020). In younger TLE patients, an association between post-operative naming decline and subtle tau hyperphosphorylation localized to only the subiculum and dentate gyrus suggest that tau-associated pathological changes in relevant brain regions over time may underlie progressive cognitive impairment seen in TLE (Prada Jardim et al., 2018). Furthermore, the neuroprotective effects of tau ablation against not only seizures but also cognitive deficits in animal models of ASD and Dravet syndrome (Gheyara et al., 2014; Tai et al., 2020) provide evidence for a role of tau in mediating cognitive impairment in these diseases as well. Therefore, tau pathology present in seizure disorders may exacerbate cognitive decline resulting from epileptic states, with seizure-driven tau hyperphosphorylation further compounding this effect with disease progression.

## Conclusion

As presented in this review, a mounting body of literature has elucidated connections between tau pathology and epilepsy disorders of diverse etiologies. The antiseizure effect of tau ablation that can be reproduced in a variety of seizure models indicates a significant mediating role of endogenous tau in epileptogenesis. Given findings of upregulated kinase and downregulated phosphatase activity across different seizure disorders, we propose that epileptic activity can trigger homeostatic responses whereby enzymatic pathways disable endogenous tau by increased phosphorylation to stabilize aberrant network hyperexcitability. Subsequent hyperphosphorylation and accumulation of tau results from overactivation of such mechanisms, especially with recurring epileptic activity, and continuous epileptic states. Furthermore, growing evidence indicates a potential contribution of tau hyperphosphorylation to progressive cognitive decline in seizure disorders. Though the exact degrees to which tau involvement in seizures and cognitive decline are mediated by convergent or divergent mechanisms in distinct diseases remains unclear, the overlapping of tau-related cell signaling pathways and prevalence of tau hyperphosphorylation found throughout different types of epilepsies (Table 1) warrant continuing efforts into understanding epilepsies from a tauopathy perspective. Greater focus on tau in epileptic pathophysiology may yield advances in diagnostic and prognostic tools and novel therapeutic approaches targeting tau and tau-associated pathways.

**TABLE 1:** Characterization of seizure disorders and their links to tau pathology and tau-associated mechanisms.

Disease	Age of onset	Symptoms	Tau pathology	Relevant signaling pathways	Potential treatment options
Temporal lobe epilepsy	All ages	Focal seizures, cognitive decline, hippocampal sclerosis	↑ total tau, ↑ p-tau, neuropil threads, pre-tangles, NFTs	mTOR, GSK-3β, PP2A	Rapamycin, lithium, sodium selenate
Post-traumatic epilepsy	Dependent on age of trauma	Seizures, cognitive decline	Acute: ↑ total tau, ↑ p-tau Chronic: ↑ p-tau, NFTs	mTOR, PP2A	Rapamycin, sodium selenate, tau reduction
Autism Spectrum disorder	Infancy to early childhood	Learning disability, anxiety and/or depression, seizures	↑ total tau, ↑ p-tau	PTEN, PI3K, mTOR, CDK5	Rapamycin, CDK5 inhibitor, tau reduction
Dravet syndrome	Infancy	Refractory epilepsy, cognitive impairment	NFTs	NMDA receptor GluN2A subunit	GNE-0273, tau reduction
Nodding syndrome	Childhood, adolescence	Seizures (vertical head nodding), cognitive and motor disability	Pre-tangles, NFTs	Unknown	
Niemann pick disease	All ages	Seizure, progressive neurodegeneration, cognitive decline	↑ p-tau, NFTs	Cholesterol, mTOR, CDK5	Rapamycin, CDK5 inhibitor
Lafora disease	Adolescence	Seizures, dementia	↑ p-tau, NFTs	Laforin, GLT-1 transporter, loss of GABAergic function	
Focal cortical dysplasia IIb	Infancy to early adulthood	Refractory epilepsy	↑ p-tau, NFTs	NMDA receptor NR2A/B subunit	CDK5 inhibitor
Tuberous sclerosis complex	All ages	Tumors, seizures, cognitive disability	↑ p-tau	mTOR, GSK-3β	Rapamycin, lithium

*CDK5, cyclin-dependent kinase 5; GSK-3β, glycogen synthase kinase-3β; mTOR, mammalian target of rapamycin; NFTs, neurofibrillary tangles; PI3K, phosphoinositide 3-kinase; PP2A, protein phosphatase 2A; p-tau, phosphorylated tau; PTEN, phosphatase and tensin homolog deleted chromosome 10.*

## Author Contributions

KV and KH developed the manuscript concept. KH prepared the figure and table. All authors contributed to the manuscript and approved the final submitted version.

## Conflict of Interest

The authors declare that the research was conducted in the absence of any commercial or financial relationships that could be construed as a potential conflict of interest.

## Publisher’s Note

All claims expressed in this article are solely those of the authors and do not necessarily represent those of their affiliated organizations, or those of the publisher, the editors and the reviewers. Any product that may be evaluated in this article, or claim that may be made by its manufacturer, is not guaranteed or endorsed by the publisher.
